# The Undeveloped Case

**Published:** 1885-01

**Authors:** J. T. Kent


### THE UNDEVELOPED CASE.

PROFESSOR J. T. KENT, A. M., M. D.

In paragraph 173, Hahnemann's *Organon*, it is strongly intimated that the "*partial* (one-sided)" case is often a hard thing to manage. It is often quite impossible to find a curative remedy where there is but one expression of the disease and that one a pathological or objective symptom. Previous symptoms have been suppressed and the only expression now in sight or obtainable is the so-called "local disease." I am asked almost every day what to do when there is a local disease and no symptoms. As a matter of fact, I seldom see such a state. But such a state may and does exist wherein there is nothing peculiar to prescribe on and there are no concomitants guiding to a remedy. The expert will nearly always see some peculiar thing in the patient to prescribe on, which prescription will generally be followed by the development of the prehistoric symptoms. If the patient can be taught to relate in his own way some old symptoms of years ago, or some symptoms that existed previous to some attack of acute disease, this may furnish a guide to a prescription that will cure or develop the latent disease. When a patient tells me that he has suffered from the present undefined "one-sided" ailment ever since he had an acute attack of any given disease, Carbo-v., very high, a single dose, will be followed after a month or two with the cure of the present disease or a development of the original true expression. Where a peculiar "one-sided" expression presents in a single symptom which is peculiar to no proved drug, it becomes necessary to develop the disease before it can be cured. Sulph. may develop symptoms that correctly express the demand for the simillimum, if not Calc. c. should follow, and in turn Lyc., always giving ample time for the last remedy to exhaust its action. Psorinum should be given on a similar absence of clear indications. This is what experience teaches in the management of latent *psora*. I have prescribed in this manner and restored many a sickly dwarf to health without ever being able to measure the obscure pathology or effect a diagnosis of his complaint. The pathological state contains very little to guide to a remedy. To prescribe Silica for *fistula in ano* is an insult to Homœopathy as much as to prescribe for pain in the stomach, diarrhœa, or headache. Who dares to say that a carcinoma could not be permanently cured with Chamomilla. When the malignant growth has come, the symptoms necessary to make a correct prescription have gone, and very little is now seen but the mass of morbid anatomy, and the *damnable* local treatment has so changed the totality that the individuality of the disease is lost, and the disease becomes masked by the pathology. The time necessary to work this case back to the prehistoric symptoms cannot be had, as the pathological evolutions are too rapid. In this case, the patient's life can only be saved when the keynote of the prehistoric identity is expressed in some two or three symptoms manifested in the morbid mass that are too persistent to be suppressed by the meddling. Such has been the case in a few instances of reported cases of cancer.

There are a few deep-acting remedies which experience has demonstrated to be useful in developing these " one-sided " cases and bringing back the original symptoms in non-malignant diseases. Sepia will often bring back the original symptoms of an intermittent fever when the case has been spoiled and the symptoms are not the true expression of the disease, because of the various remedies prescribed inappropriately. These "one-sided" cases seldom occur primarily. By this I mean that when a disease has not been treated inappropriately, it will express itself in the features of a perfect and natural picture, generally known and recognized by every hard-working prescriber.

It is commonly spoken by the superficial observer that a localized inflammation is a local disease *per se*, because there are no symptoms of a constitutional disease. It is a very common thing to see this so-called local disease alternate with cough, pain in the back, and other circumscribed evidence of *psora;* and still this superficial observer learns no lesson and continues in his deception, for such it is, and a double one, as he deceives both himself and his patient. To treat these " one-sided " diseases, it is necessary to take the symptoms of each separate manifestation in perfect expressions, and the totality will express the individuality of the disease. These separate and distinct localizations may be years apart and there exist a long period of so-called health. So far apart are these one-sided expressions that they are not supposed to be connected unless the physician is conversant with all the peculiar habits of *psora*. In an artificial way we have an example of a one-sided disease in the seton. I have seen a host of symptoms depart soon after the issue began to suppurate. I might as well prescribe for that seton as to prescribe for the disease in the absence of its own natural expressions. Would any sane man call this a issue local disease? Not long ago I was treating a man for stomach symptoms. He had always been treated in the old way and it was with difficulty that I could urge him to express his symptoms. Finally I gave him entire relief from his so-called dyspepsia, and behold! he got conjunctivitis with paralysis of the lid of the left eye, with dimness of vision as though looking through a gauze. Causticum corresponds to the eye symptoms and the stomach symptoms which I supposed I had cured, and cured permanently. One of our St. Louis oculists had suppressed this conjunctivitis four times, believing that it was a local disease. The importance of including the cured symptoms in the last remedy is quite visible. If this is not done the old symptoms may return. One fortunate thing is, that the new symptoms usually correspond to a remedy having the old ones. However, if the choice is difficult the old symptoms are a great help.

Over a year ago a gentleman consulted me, saying that he was rapidly losing his eyesight. He had changed glasses several times and the oculist had failed to benefit him, and he feared blindness. He had been forced to give up his business, which was bookkeeping. Nothing about the eye revealed the cause of weak sight. The rest, therefore, had not helped him. He complained of no symptoms at present, but ten years previously he suppressed a chill by quinine.

The symptoms of that attack seemed to me like Carbo-v. and I had nothing else to base a prescription on, as there was no guiding symptoms in the threatened blindness. He got Carbo-v., 76m Finke, one dose, and immediate improvement followed in his vision. He took a violent cold that developed Phosphorus symptoms in the chest, and one dose of that remedy, 5m, restored him to health and also finished the case as to his sight. Another evidence of the complementary relations of Carbo-v. and Phos. in undeveloped cases.

Disease of psoric nature may present alternating manifestations where any one of the manifestations may be considered by the superficial observer as a distinct acute disease. Diarrhœa may exist for several days and stop suddenly and a rheumatism come on. While the characteristics are not indications of the remedy in either of these expressions, this peculiar order of expression will nearly always find its remedy in *Abrotanum.* It is often a difficult matter to prescribe correctly on the one or two symptoms found in the localized expression, but by an appropriate prescription for the "one-sided" expression, the other side may develop, and the new, necessarily changed, prescription, including the old symptoms, will most likely change the alternation habit and cure or develop simpler expressions of the disease.

Thus we trace back to the original or primary simple disease a complexity of symptoms that none but the experienced should attempt to treat. Incautious prescribing, repeating medicine in inappropriate potencies, can never unravel such cases. Every dose must be permitted to finish its action, as very often it is necessary to secure the effect of the secondary action of a given remedy in the finest detail of its tapering-off properties. It will often be observed with Calc., Sulph., Graph., and all of that class of remedies, that the highest order of curative effect is secured in the fourth, fifth, and sixth weeks, and even much later.

To understand these "one-sided" disease expressions and be successful in their management, the physician must accept as truth that they are all constitutional and of psoric origin. He who looks upon them as local disease is a curse to such patients as he is permitted to treat. Every time a so-called local disease is driven back, it is that much harder to reach with correct prescribing.

It is a sorry fact that many such men claim to be homœopathists, and do untold injury to the system of medicine they so poorly comprehend and so imperfectly follow.

